# Arthropod prey type drives decomposition rates and microbial community processes

**DOI:** 10.1128/aem.00394-24

**Published:** 2024-06-25

**Authors:** Jessica R. Bernardin, Sarah M. Gray, Leonora S. Bittleston

**Affiliations:** 1 Department of Biological Sciences, Boise State University, Boise, Idaho, USA; 2 Department of Biology-Ecology and Evolution, University of Fribourg, Fribourg, Switzerland; Georgia Institute of Technology, Atlanta, Georgia, USA

**Keywords:** microbial communities, bacterial function, decomposition, pitcher plant, hydrolytic enzyme activity, *Sarracenia*

## Abstract

Microbial communities perform various functions, many of which contribute to ecosystem-level nutrient cycling via decomposition. Factors influencing leaf detrital decomposition are well understood in terrestrial and aquatic ecosystems, but much less is known about arthropod detrital inputs. Here, we sought to infer how differences in arthropod detritus affect microbial-driven decomposition and community function in a carnivorous pitcher plant, *Sarracenia purpurea*. Using sterile mesh bags filled with different types of sterile arthropod prey, we assessed if prey type influenced the rate of decomposition in pitcher plants over 7 weeks. Additionally, we measured microbial community composition and function, including hydrolytic enzyme activity and carbon substrate use. When comparing decomposition rates, we found that ant and beetle prey with higher exoskeleton content lost less mass compared with fly prey. We observed the highest protease activity in the fly treatment, which had the lowest exoskeleton content. Additionally, we saw differences in the pH of the pitcher fluid, driven by the ant treatment which had the lowest pH. According to our results from 16S rRNA gene metabarcoding, prey treatments with the highest bacterial amplicon sequence variant (ASV) richness (ant and beetle) were associated with prey that lost a lower proportion of mass over the 7 weeks. Overall, arthropod detritus provides unique nutrient sources to decomposer communities, with different prey influencing microbial hydrolytic enzyme activity and composition.

**IMPORTANCE:**

Microbial communities play pivotal roles in nutrient cycling via decomposition and nutrient transformation; however, it is often unclear how different substrates influence microbial activity and community composition. Our study highlights how different types of insects influence decomposition and, in turn, microbial composition and function. We use the aquatic pools found in a carnivorous pitcher plant as small, discrete ecosystems that we can manipulate and study independently. We find that some insect prey (flies) breaks down faster than others (beetles or ants) likely because flies contain more things that are easy for microbes to eat and derive essential nutrients from. This is also reflected in higher enzyme activity in the microbes decomposing the flies. Our work bridges a knowledge gap about how different substrates affect microbial decomposition, contributing to the broader understanding of ecosystem function in a nutrient cycling context.

## INTRODUCTION

Microbial communities drive global nutrient cycling. Microbes cycle simple and complex nutrients that then can be used by other unicellular or multicellular food web members. The contributions microbial communities make to decomposition and nutrient cycling are dynamic and complex, and unique biotic and abiotic conditions selected for compositionally and functionally optimized decomposer communities. One of the components most likely to affect heterotrophic microbial function is the nutrient composition and digestibility of the input substrate. In terrestrial experiments, manipulations of leaf litter or deposition of nutrients correlate with changes in microbial community composition (1). For example, the stimulation of microbial growth by high-quality litter showed that nutrient input has differential effects on microbial growth and decomposition (2). The chemistry and species composition of plant litter are known to directly impact the rate at which it decays, influencing the microbially produced extracellular enzymes and the accumulation of microbial biomass (3–5). Here, we expand on this by investigating how different species of arthropod detritus influence both community and ecosystem-level processes.

Microbial community composition and function are driven by biotic and abiotic factors, with influences from light, time, pH, temperature, litter type, and more (6–9). In fact, microbial communities have been found to moderate volatile organic compound (VOC) emissions in litter experiments (10), shift across time to influence leaf litter decomposition via changes in enzyme production (11, 12), and show spatial patterns in how and what nutrients are translocated (13). Additionally, the litter “home-field advantage hypothesis (HFA)” postulates that litter decomposes faster in the vicinity where it originated due to the presence of specialized decomposers (14). A meta-analysis found evidence of a worldwide HFA; they saw a 7.5% faster decomposition at home, which became stronger when the home and away litters were more dissimilar (15). These results emphasize the importance microbial communities play in decomposition and nutrient cycling, and the complexity of factors influencing their composition and function (16).

Terrestrial and aquatic systems experience episodic and seasonally dependent pulses of resources in the form of plant and carrion detritus, which can be defined as any type of dead organic matter (17). Carrion detritus, although often considered less abundant, has direct impacts on ecosystem processes, such as the case of lake or marine snow from zooplankton (18, 19). In a more specific example, deposition of cicada detritus into freshwater streams and ponds impacted the stability of food web functional groups (20). Generally, the literature exploring microbial nutrient cycling in terrestrial systems is centered around leaf litter detrital inputs, but less is known about how arthropod type and quality relate to decomposition and microbial community functions, despite terrestrial arthropods being a major component of food webs (21). Just like plant litter, arthropods differ in their chemical composition and physical properties, with distinct differences in biomass, surface area, and chemical diversity (22–27).

The carnivorous purple pitcher plant, *Sarracenia purpurea,* offers a unique opportunity to test how arthropod substrates affect microbial function and composition in a small, natural, freshwater system under semi-controlled conditions. The microbial communities that develop inside purple pitcher plants rely on the plant to capture arthropod prey, and the plant relies on the microbial community it hosts to break down that prey and release nutrients that are severely limited in the soils in which it grows (28–30). Evidence from a recent study showed that the presence or absence of prey additions affected microbial community functions like hydrolytic enzyme activity, including chitinase and protease enzymes, which in turn were linked to the transformation of insect nutrients into more plant-available forms (31). However, they did not test different prey types, and it is still an open question how prey type influences microbial function in this system. This interplay between microbes, insects, and plants provides a framework to experimentally test how differences in arthropod prey influence decomposition and microbial community function and composition.

Pitcher plants have cup-shaped leaves, which fill with rainwater and trap arthropods including ants, flies, bees, beetles, and more (32, 33). In addition to providing nutrients, insect prey provides increased habitat variability: increasing surface area and niche space for microbial decomposers. The microbes in these fluid-filled pitchers contribute to nutrient cycling in two main ways. First, the nutrients released from arthropods are part of a microbially mediated degradation process by which larger microbial community members shred prey while bacterial microbes secrete hydrolytic enzymes that mineralize complex organic matter into smaller molecules that can be absorbed by the pitcher. Second, insect prey provides excess energy and nutrients important for microbial growth and metabolism, which in turn influences the abundance and diversity of the microbial community.

*Sarracenia purpurea* subsp. *purpurea* was introduced into Switzerland (Jura mountains and Canton de Vaud) in the 19th century (34). These plants have naturalized in peat bogs and lowland wetlands, growing prolifically (35). In contrast to the *S. purpurea* found in North America, the food web is less complex in its Swiss counterparts, with membership limited to bacteria, fungi, protozoa, and mites (32, 33, 36). Because of the absence of midge, mosquito, and flesh fly larvae, prey decomposition does not go through the shredding process observed in pitchers found in their native range.

**Fig 1:**
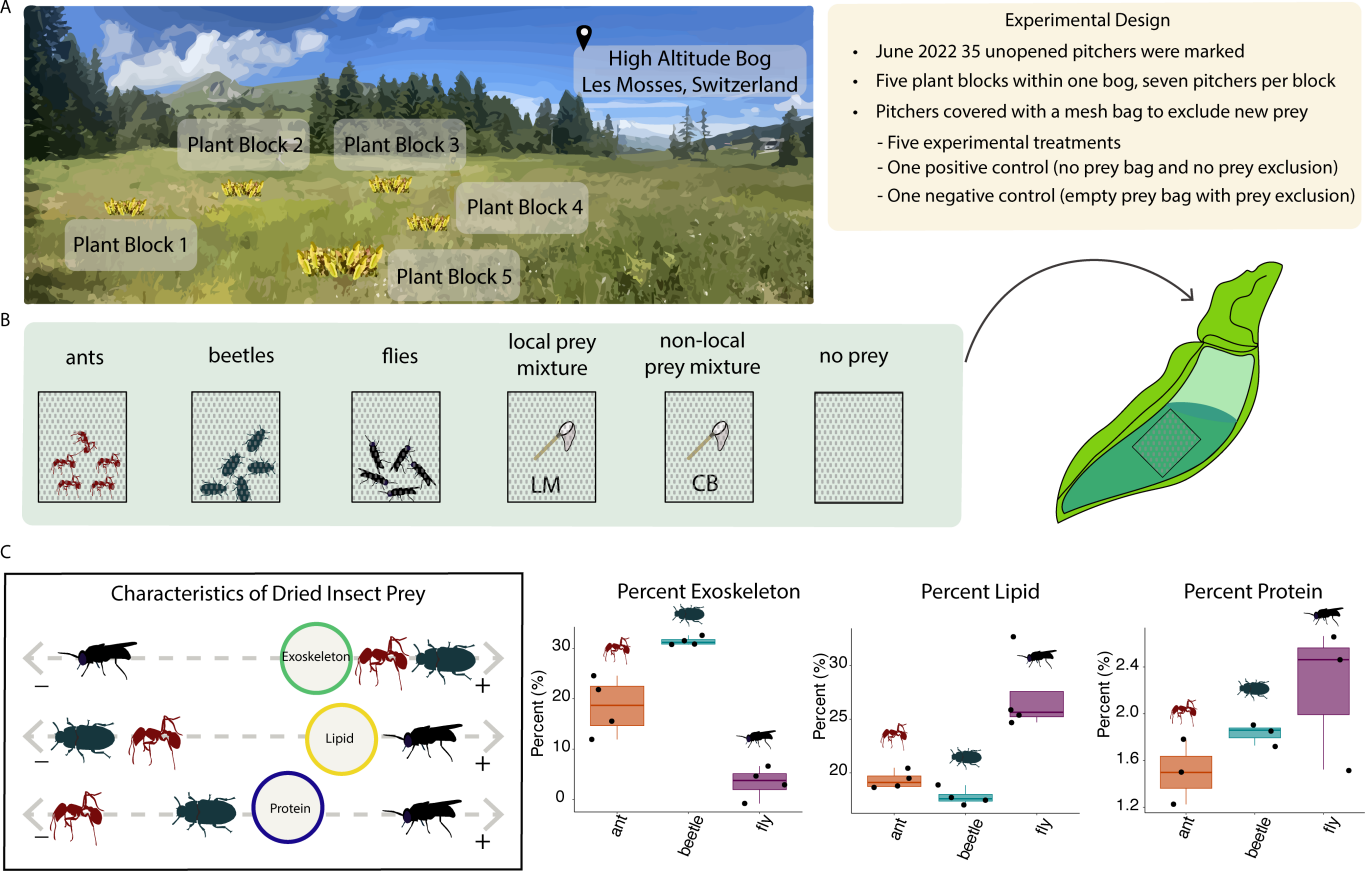
A schematic figure illustrating the experimental design and nutritional differences in insect prey. (**A**) Seven unopened pitchers were selected from each of five plant blocks in a high-altitude Swiss bog. (**B**) At day 0, prey bags were added to each pitcher (one replicate of each treatment per block) and pitchers were covered with a fine mesh prey exclusion bag (except for positive control, not shown). (**C**) We characterized nutrient composition for the single-species prey (ants, beetles, and flies). Flies had the highest proportion of lipid content and the lowest exoskeleton content, whereas beetles and ants had the highest exoskeleton content.

Our primary objective was to determine what effect prey type has on community-level processes—like microbial community assembly and function—and larger-scale processes, such as decomposition, in this naturalized population of *S. purpurea* in Switzerland. We addressed this question by adding Diptera, Hymenoptera, and Coleoptera prey, which differed in their macronutrient composition, to the leaves of *S. purpurea* in a field experiment (Fig. 1). We then followed their decomposition rates and associated microbial community composition and function through time. We included both local and non-local prey treatments to determine to what effect “home field advantage” and resource complexity influenced microbial function. We hypothesized that (1) prey type modulates ecosystem-level processes such as prey decomposition in pitcher plant environments. More specifically, we hypothesized that prey with a higher exoskeleton (composed mostly of chitin) percentage of total body mass would have decreased decomposition compared with prey composed of higher levels of proteins or lipids that are easier to access and metabolize by microbes. Additionally, we expected that arthropod prey type would shape microbial function and composition in pitcher plant systems. Thus, we hypothesized that (2) prey type would impact microbial functioning in terms of specific metabolic processes (i.e., enzyme activity, carbon substrate use) and overall microbial community characteristics (abundance of living cells and pH). Additionally, we hypothesized that (3) different prey would lead to discernible differences in the microbial community composition within these aquatic ecosystems. Finally, we hypothesized that (4) microbial communities exposed to local prey would have a “home-field advantage” and show an increased decomposition rate and increased microbial functional activity compared to non-local prey.

## RESULTS

### Prey composition

At the start of the experiment, the prey bags were standardized across treatments by biomass. The ant, beetle, and fly prey bags differed in terms of the proportion of exoskeleton and lipids, but not proteins (Fig. 1C; Fig. S1). We did not have enough local and non-local prey to do the required number of replicates for nutritional compositional assays, but these treatments were composed of a mixture of insects caught at each site, local Les Mosses (LM), and non-local Champ Buet (CB). For the local prey, the types of prey were balanced between replicates within the same treatment and contained a mixture of locally caught ants, beetles, flies, grasshoppers, spiders, hemipterans, and lepidopterans. For the non-local prey, prey types were also balanced between replicates within the same treatment and contained a mixture of non-locally caught ants, beetles, flies, grasshoppers, spiders, and hemipterans. Additionally, the CB site had spiders that were much larger (~43% of dry biomass of total prey caught) than the LM site (~ 11%); hence, the proportion of spider mass in the CB bags was higher.

### Prey decomposition

**Fig 2:**
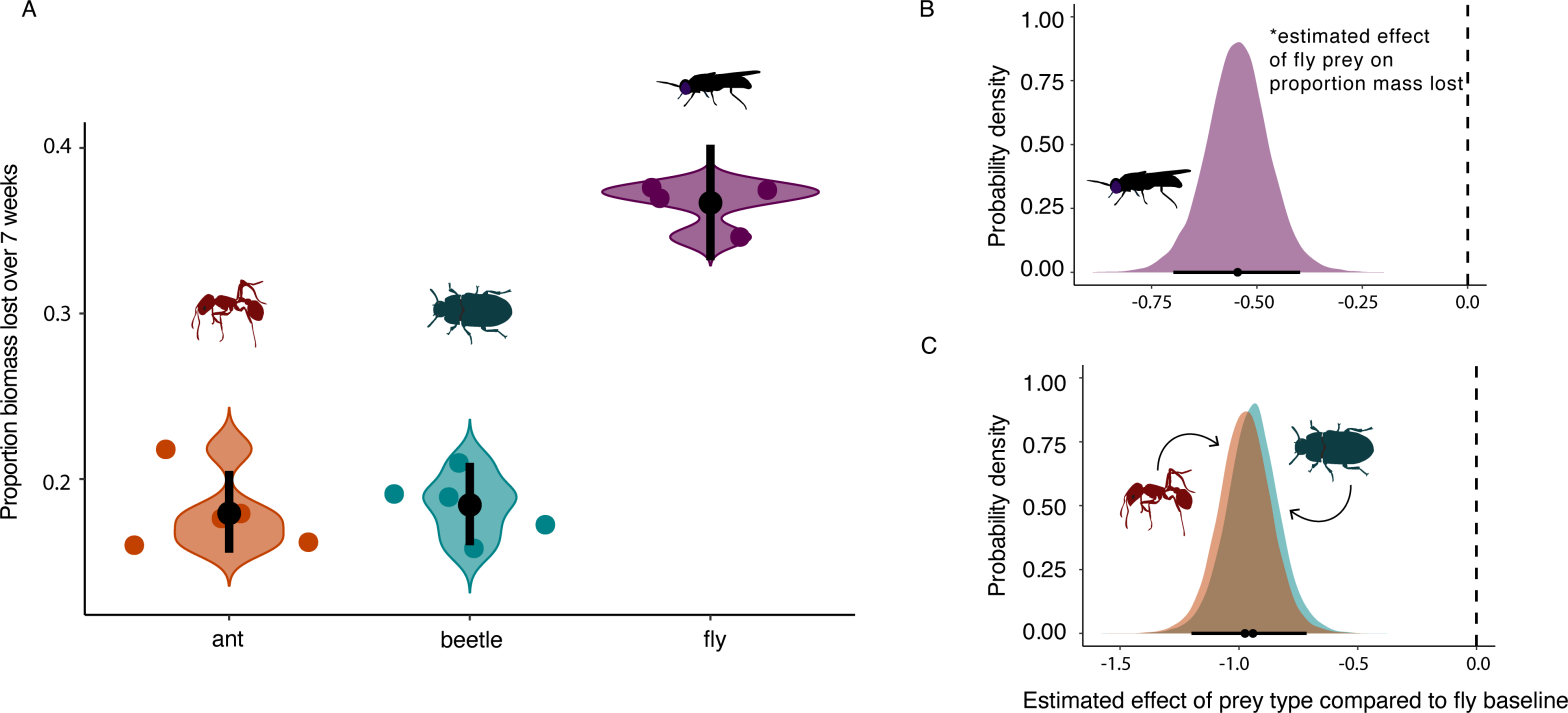
Prey decomposition rates depend on prey type. (**A**) The proportion of mass lost over 7 weeks for the different types of insect prey inside mesh bags. The colored points represent individual samples, and the black points represent the estimated marginal effects along with 95% credibility intervals for each estimate. (**B**) The posterior estimate of the proportion of mass lost for fly prey bags. (**C**) The posterior estimates of the proportion of mass lost for each treatment compared with fly (baseline, 0). The points represent the median estimate, and the black bars represent the 95% credibility intervals around those estimates.

Fly prey bags lost approximately twice the biomass compared with ant and beetle prey bags, supporting hypothesis 1 (Fig. 2A). The estimated mass lost by decomposition from the ant and beetle prey bags was 17.9% and 18.4%, respectively (95 CIs_ant_ = 0.16, 0.20; 95 CIs_beetle_ = 0.16, 0.21, CI = credibility intervals), whereas it was 36.6% in the fly prey bags (95 CIs = 0.33, 0.40). This is also reflected in the posterior estimates from our model (Fig. 2B and C).

### Microbial functioning

**Fig 3:**
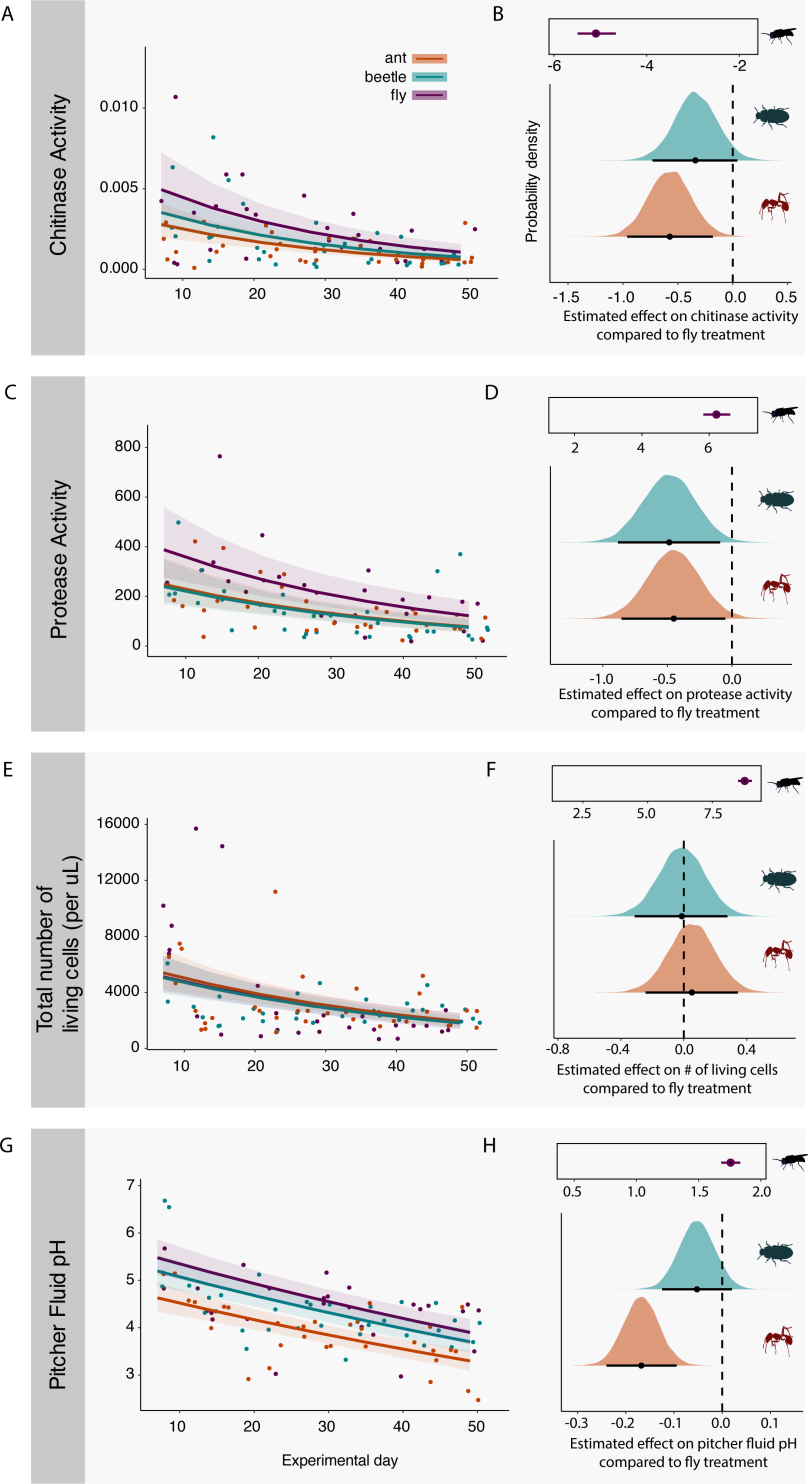
Microbial functioning across prey treatments. For the left-hand panels, the points show the measures of each variable from each pitcher through sampling time, and the line represents the median marginal effects and 95% credibility intervals over time. The right-hand panels show the corresponding posterior density estimates, with estimates in the black boxes representing the model intercept (fly prey), which is the baseline estimate (0) to which the parameter estimates are compared. In all cases, the black point represents the median estimated effect compared with the baseline with all other parameters held at their means, the black bar represents the 95% credibility intervals, and the density plot shows the distribution of the posterior draws. The x-axes represent values calculated from the log link function. (**A**) Chitinase activity (µg substrate/min) measured for each pitcher fluid sample weekly from day 7 to day 49. (**B**) Posterior density estimates of prey type on chitinase rate. (**C**) Protease activity (ng substrate/min) measured for each pitcher fluid sample weekly from day 7 to day 49. (**D**) Posterior density estimates of prey type on protease rate. (**E**) Bacterial living biomass (living bacterial cells per µL pitcher fluid) measured for each pitcher fluid sample weekly from day 7 to day 49. (**F**) Posterior density estimates of prey type on living cells. (**G**) Pitcher fluid pH measured for each pitcher fluid sample weekly from day 7 to day 49. (**H**) Posterior density estimates of prey type on pitcher fluid pH.

To investigate if the prey treatments influenced specific functions and community conditions (hypothesis 2), we built generalized linear mixed models (GLMMs) for each response of interest: chitinase activity, protease activity, living bacterial cells, and pitcher fluid pH. In general, chitinase, protease, and living bacterial cells decreased over time (Fig. 3), and there were several differences between prey treatments.

Specifically, the median chitinase rate for the fly treatment was 0.0023 µg/min (95 CIs = 0.0018, 0.0032), and this is statistically higher than the estimated chitinase rate in the ant treatment (0.0013 µg/min, 95 CIs = 0.001, 0.002) but not different from beetle prey (0.0017 µg/min, 95 CIs = 0.0013, 0.0021, Fig. 3A and B). For protease activity, the fly treatment was also highest (Fig. 3C), with statistically lower protease activity in both the ant and beetle treatments (Fig. 3D). The fly prey treatment had a median estimate of 219 ng/min (95 CIs = 165.8, 298.1), the ant prey had the second lowest estimate of protease rate at 140 ng/min (95 CIs = 108.5, 184.1), and the lowest rate was observed in the beetle treatment at 135 ng/min (95 CIs = 104.5, 178.2).

In terms of bacterial density, the three prey treatments did not differ in numbers of living cells (Fig. 3E). Fly treatments had an estimated 3,106 living cells/µL of pitcher fluid (95 CIs = 2,519; 3,888, Fig. 3F). Numbers of living cells tended to be lower in ant and beetle prey, although 95% credibility intervals crossed 0, and ranged from an estimated 3,270 living cells/µL (95 CIs = 2,717; 3,973) in the ant treatment to 3,067 cells/µL (95 CIs = 2,555; 3,735) in the beetle treatment (Fig. 3F). The pH of pitcher fluid (Fig. 3G) from the ant prey treatment was the lowest of all the treatments with an average pH of 3.9 (95 CIs = 3.7, 4.1, Fig. 3H), compared with pH 4.6 in the fly treatment (95 CIs = 4.4, 4.9) and pH 4.4 in the beetle treatment (95 CIs = 4.2, 4.6).

**Fig 4:**
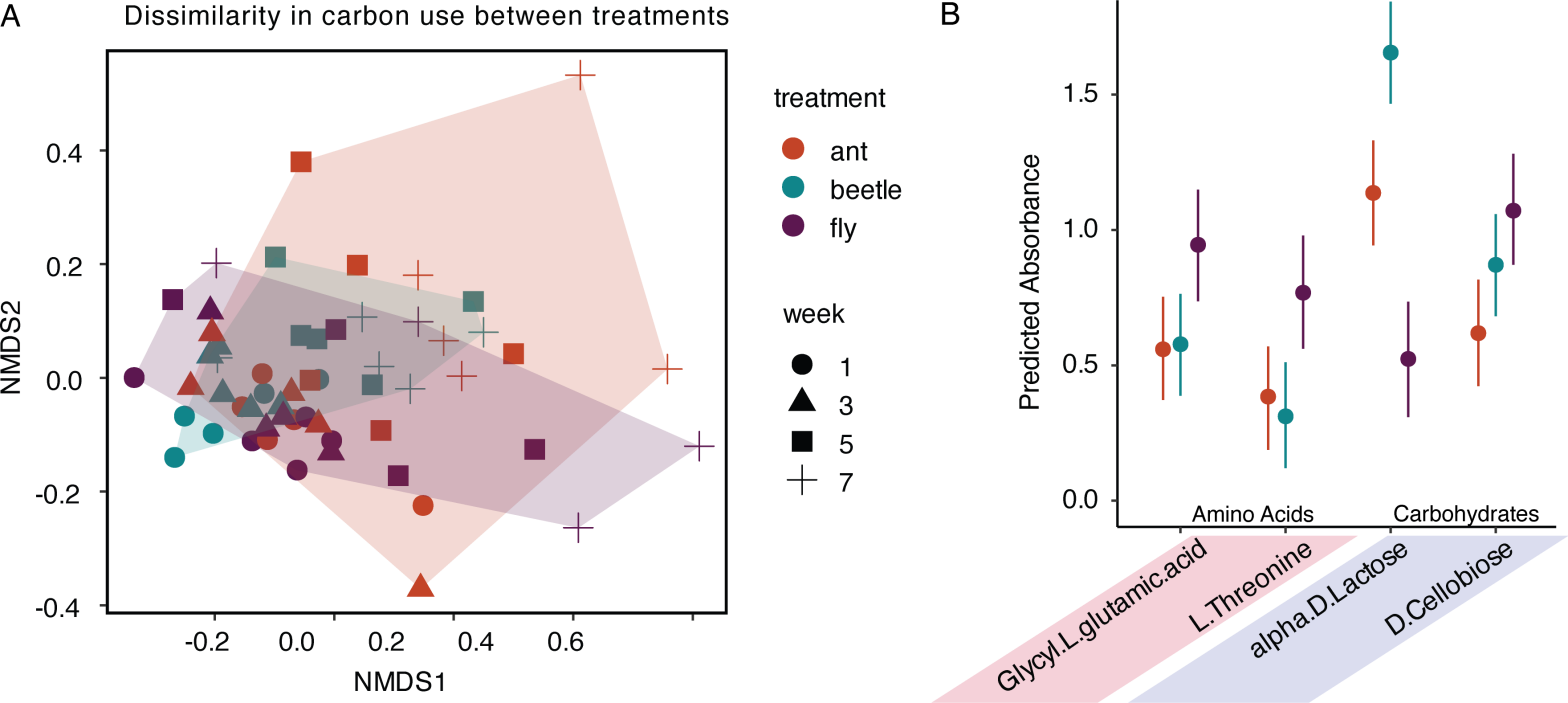
Carbon substrate use for ant, beetle, and fly prey treatments. (**A**) Non-metric multidimensional scaling (NMDS) of Bray-Curtis dissimilarities between carbon substrate used for microbial samples across ant, beetle, and fly prey treatments at four time points (k = 2, stress = 0.16), colored by treatment (PERMANOVA; R^2^ = 0.07801, F_4,87_=2.4858, *P* = 0.003) and time (PERMANOVA; R^2^ = 0.24018, F_3,87_=10.2045, *P* = 0.001). (**B**) Four carbon substrates showed statistically different carbon substrate metabolism between at least two of the three prey treatments. Point represents the estimated marginal effect of substrate on absorbance, and the vertical bar represents the 95% credibility intervals around each estimate.

We assessed carbon use dynamics between our treatments through time using EcoPlates. Nonmetric multidimensional scaling (NMDS) ordination based on Bray-Curtis dissimilarities showed overlapping points among treatments, with shifts in carbon use over time (Fig. 4A). We measured no differences in dispersion (37) by treatment (F_2, 54_=1.8294, *P* = 0.1703), but significant differences in dispersion through time (F_3, 53_=4.6998, *P* = 0.006). Using a permutational multivariate analysis of variance (PERMANOVA) to test for differences in carbon use by prey treatment and through time, we found significant effects of treatment (PERMANOVA; R^2^ = 0.07801, F_4,87_=2.4858, *P* = 0.003) and time (PERMANOVA; R^2^ = 0.24018, F_3,87_=10.2045, *P* = 0.001). We found that treatment groups accounted for approximately 7% of the variation in our EcoPlate data, whereas time accounted for 24%. A post-hoc pairwise analysis between treatments found significant differences in substrate use profiles between all treatments (ant vs beetle, fly vs ant, and ant vs fly, Table S2). We tested for differences in the use of specific substrates between the beetle and ant prey treatments compared with the fly prey (Fig. S2). Our model predicted that the bacteria in the fly treatment had lower metabolic capacity for the carbohydrate lactose compared with beetle and ant prey. Additionally, the bacteria in the fly treatments showed increased metabolism in the substrates glycyl glutamic acid, threonine, and cellobiose (Fig. 4B).

### Microbial composition

To address hypothesis 3, we measured the microbial community composition of the pitcher fluid at three time points over 7 weeks (days 7, 14, and 35). After quality control (removing contaminants and non-prokaryotic ASVs) and rarefaction, we recovered 441 ASVs across the 98 samples (5 prey treatments + 2 control treatments × 4–5 pitchers per treatment × 3 time points). Within our samples in the ant, beetle, and fly treatments, we identified 11 bacterial phyla, 57 families, and 88 genera. The most abundant genera were: *Pedobacter* (23.0%), *Pseudomonas* (19.7%), and an uncultured genus in the Family Chitinophagaceae (8.8%) (Fig. S3).

**Fig 5:**
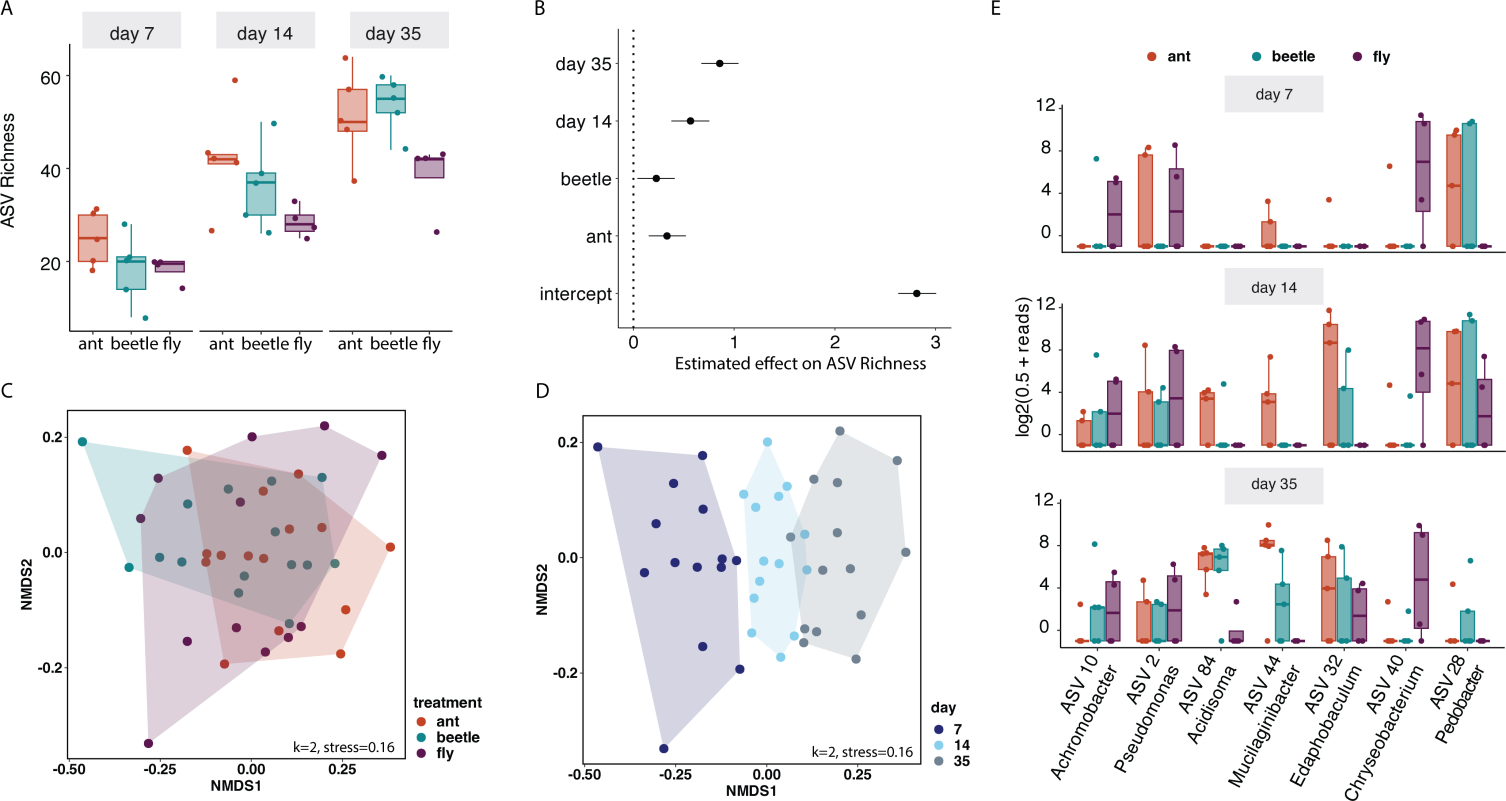
Microbial composition between the ant, beetle, and fly treatments. (**A**) ASV richness across days 7, 14, and 35 for the three prey treatments. (**B**) Posterior probability estimates for ASV richness based on treatment and time. The black point is the median estimate, and the black bars represent the 95% credibility intervals (based on a GLM with a negative binomial distribution). (**C, D**) Non-metric multidimensional scaling (NMDS) of unweighted UniFrac distances for microbial samples across ant, beetle, and fly prey treatments at three time points (k = 2, stress = 0.16), colored by treatment [C, PERMANOVA Df_2,37_, R^2^ = 0.22, *P* < 0.001)] and time (**D**). (**E**) Seven differentially abundant ASVs (subset from the 13 taxa identified) at days 7, 14, and 35. Reads were transformed by log2(0.5 + reads), both ASV number and genus name are listed on the x-axis. In all cases, boxplots represent the interquartile range (IQR) of the counts for each sample in each treatment, and the whiskers extend 1.5 times the IQR, with the horizontal bars representing the medians.

We observed differences in ASV richness between our treatments and through time (Fig. 5A and B). We estimated that the fly treatment had a mean richness equal to 16.7 ASVs (95 CIs = 13.9, 20.2), whereas the beetle and ant treatments had statistically higher richness at 20.9 (95 CIs = 17.6, 24.8) and 23.3 (95 CIs = 19.7, 27.6), respectively. Additionally, we saw an independent effect of day on richness (Fig. 5B) with an increase in richness across all three treatments over time.

Based on unweighted UniFrac distances, we observed differences in community composition between bacterial communities (Fig. 5C, PERMANOVA Df_4,62_, R^2^ = 0.09, *P* = 0.001). Pairwise comparisons found compositional differences between the ant vs fly treatments, but not between ant vs beetle or beetle vs fly (Fig. 5C; Table S3). Likewise, we found differences in bacterial community composition through time (Fig. 5D, PERMANOVA Df_2,62_, R^2^ = 0.21, *P* = 0.001). Similar to the patterns we saw in our carbon substrate use analysis, we found that time accounted for a higher percentage of the variation (21%) compared with treatment (9%).

We investigated if any ASVs were differentially abundant across pairwise comparisons of our three insect prey treatments. Using an analysis of compositions of microbiomes with bias correction (ANCOM-BC), we identified 13 differentially abundant ASVs (Fig. S4), with the seven ASVs that had the clearest differences shown in Fig. 5E. All were from different genera, and *Chryseobacterium* ASV 40 had much higher abundance in the fly samples, whereas *Mucilaginibacter* ASV 44 had consistently higher abundance in ant samples (Fig. 5E).

Overall, protozoan abundance was very low, and we found no difference in the alpha or beta diversity of protozoa between our treatments (Fig. S5). In terms of protozoan richness, we saw no statistical differences between the three treatments. Thus, the differences in bacterial richness did not alter the dynamics of their predators (protozoa). On average, morphotype richness was less than one in all cases. We found no differences in dispersion between treatments (F_6,110_=1.293, *P* = 0.2664) and no differences in protozoan community composition based on prey treatment (PERMANOVA; R^2^ = 0.05231, F_6,103_=1.0309, *P* = 0.074).

### Local vs non-local prey

**Fig 6:**
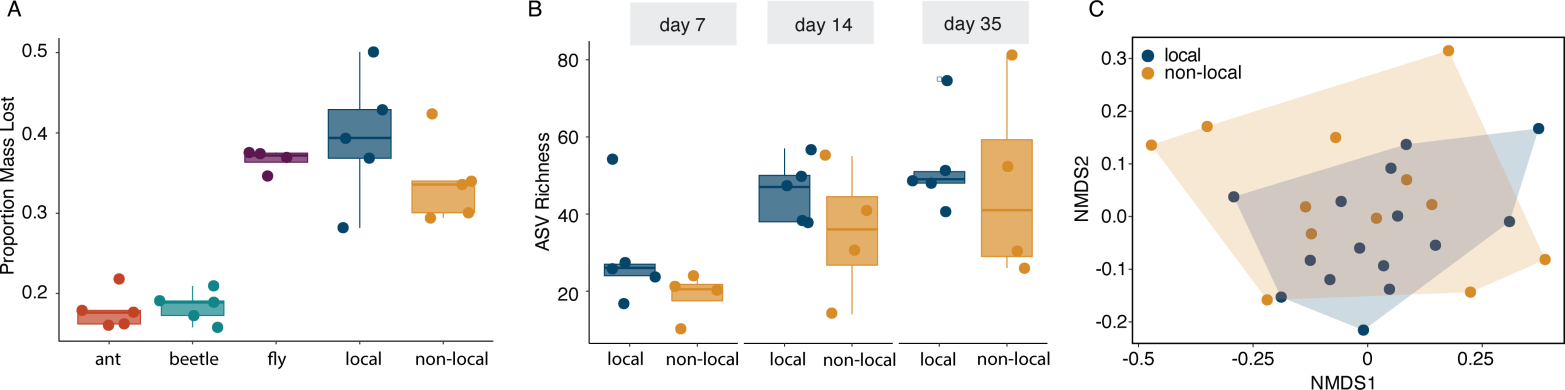
Local versus non-local prey show very similar trends. (**A**) The proportion of mass lost for the different arthropod prey over 7 weeks *in planta*. (**B**) Bacterial richness in pitcher fluid that had local vs non-local prey at day 7, day 14, and day 35. For (**A and B**), boxplots represent the IQR of the values for each sample in each treatment, and the whiskers extend 1.5 times the IQR, with the horizontal bars representing the medians, and points being the sample values. (**C**) Nonmetric multidimensional scaling (NMDS) of unweighted UniFrac distances for microbial samples across local and non-local prey treatments at three time points (k = 2, stress = 0.116), colored by treatment (PERMANOVA Df_1,23_, R^2^ = 0.088, *P* = 0.045).

We hypothesized that microbial communities exposed to local prey would have a “home-field advantage” and show an increased decomposition rate and increased microbial functional activity compared with non-local prey (hypothesis 4). However, we found no differences in the decomposition between local and non-local prey. The estimated mass lost by decomposition was 39% and 34%, respectively (95 CIs_local_ = 0.32, 0.47; 95 CIs_non-local_ = 0.27, 0.41), which was similar to that found in the fly treatment at 37% (Fig. 6A, Fig. S6). However, we did observe higher decomposition in local, non-local, and fly prey compared with the ant and beetle treatments (Fig. 6A). We observed no differences in microbial function (chitinase, protease, living bacterial cells, pH, carbon substrate use) between our local and non-local prey (Fig. S7). For ASV richness, we observed no differences between our treatments, but we did observe a weak positive effect of time (Fig. 6B; Fig. S6). We estimated that the local treatment had a mean richness equal to 38 ASVs (95 CIs = 31.0, 47.5), and the non-local prey had a mean richness of 29 (95 CIs = 23.0, 37.9). Additionally, we see an independent effect of day on richness (Fig. 6B; Fig. S6) with an increase in richness across both treatments through time. Based on unweighted UniFrac distances, we did observe a small difference in community composition between bacterial communities in the local vs non-local treatments (Fig. 6C, PERMANOVA Df_1,23_, R^2^ = 0.088, *P* = 0.045). There were no differentially abundant taxa identified between the two prey types.

## DISCUSSION

### Building a conceptual model of insect detrital decomposition

**Fig 7:**
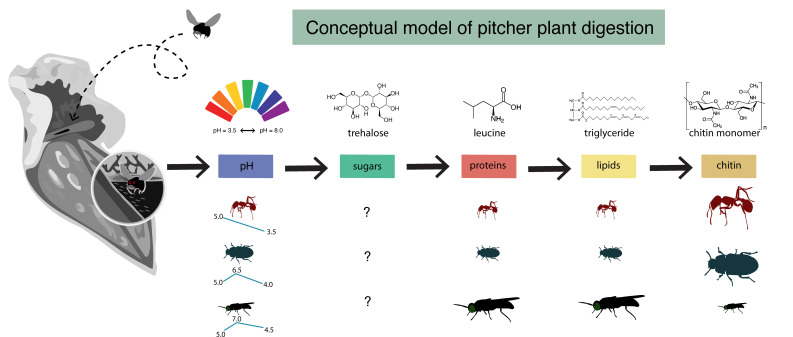
A conceptual model for arthropod digestion in the pitcher plant system. Arthropod type influences the pH of pitcher fluid. Easy-to-metabolize substrates like carbohydrates (sugars) are consumed first, helping to establish a growing microbial community. Then, microbes produce hydrolytic enzymes that transform proteins and lipids into more bioavailable sources of nitrogen that become a common good for other members of the microbial community and the pitcher plant. Lastly, chitin, which is more recalcitrant, offers an important source of nitrogen to the system at the end of the decomposition process.

Microbial communities perform various functions, many of which contribute to ecosystem-level nutrient cycling via decomposition and nutrient transformation. Factors influencing detrital decomposition are well understood in terrestrial and aquatic ecosystems, but much less is known about arthropod detrital inputs, especially in freshwater ecosystems. Here, we sought to infer the relationship between differences in arthropod detritus and microbial-driven decomposition and related functions. By comparing decomposition and microbial functions across three unique prey types, we showed that detrital type has differential effects on decomposition, microbial hydrolytic enzymes, and microbial substrate use. We propose a conceptual model for detrital decomposition in pitcher plants (Fig. 7), in which prey drives pitcher fluid pH, and less complex substrates like sugars are metabolized more quickly than more recalcitrant substrates like chitin. Furthermore, we expect that prey that vary in nutrient composition will alter the speed at which microbial communities transition through this process. For example, microbial communities digesting protein-rich prey will likely have increased protease activity compared with those communities digesting prey with lower protein content. Additionally, each new prey addition adds a new pulse of nutrients that enable microbes to be more efficient at metabolizing particular substrates, similar to what we see in the positive control (open) pitchers with peaks in functional activity occurring later in time (Fig. S7).

### Like litter, arthropod decomposition rates are input dependent

We assessed if prey type influenced the rate of decomposition (proportion mass lost) inside purple pitcher plants over 7 weeks (Fig. 2). When comparing the decomposition rates of ant, beetle, and fly prey, we found that ant and beetle prey had distinctly lower decomposition compared with flies, supporting our hypothesis (H1) of a relationship between prey nutrient composition and the proportion of mass lost: prey with higher exoskeleton content lost less mass than prey with higher lipid levels. Interestingly, the source location of the prey type (local vs non-local) did not impact decomposition or microbial processes (hypothesis 4). Instead, the mixed-species prey from both source locations lost the same proportion of mass as the fly prey alone. This may be due to the fact that flies were the dominant prey captured at both sites and are more generally known to be the dominant prey in Switzerland (33). The absence of support for the HFA hypothesis in our study could potentially be due to the sterilization of prey, removing a source of locally adapted microbes. However, we think it is more likely to be due to the diversity and ease of decomposition of prey captured at both sites. Recent work testing HFA found the lack of support for HFA when litter was easy to decompose (more labile) and support for HFA when litter was more difficult to decompose (more recalcitrant) (38).

A pattern similar to our results has been observed in leaf decomposer communities, where leaf chemistry influenced not only leaf decomposition rates but also microbial function, with unique functional guilds assembling depending on substrate type (4). Likewise, there is evidence that more recalcitrant litter types (e.g., lignin) have negative relationships with decomposition rates with litter quality (e.g., the contents of N, P, K, Ca, Mg, and the C:N and lignin:N ratios) being the most important regulator of decomposition on the global scale (39). In our case, the most recalcitrant nutrient in insects is likely to be chitin, which is the main component of the exoskeleton in arthropods. Our results show that prey with higher exoskeleton content (beetle and ant) have decreased decomposition in 7 weeks compared with prey with lower proportions of exoskeleton and higher lipid content (fly).

### Different effects are found across microbial functions

Our results provide mixed evidence for the impacts of prey type on microbial function, suggesting the complexity of functional dynamics is sometimes difficult to capture in natural microbial communities. Supporting hypothesis 2, we observed the highest protease activity in the fly treatment, which had the highest average percent protein, although it was not statistically different from the other two prey types (likely due to being underpowered from having only 3 samples per treatment). We expected the prey with the higher exoskeleton content (ant and beetle) to have increased chitinase activity, but instead, the fly treatments had the highest chitinase activity, then beetle, and then ant was the lowest. A possible explanation could be that chitinase dynamics happened earlier in the experiment (before day 7) and was not captured by our sampling. A previous experiment in the pitcher plant system found differences in hydrolytic enzyme dynamics across time, with peaks in activity occurring between 6 and 48 hours after nutrient addition; however, that study used substrates that are easier to metabolize (fruit flies, glucose, and glutamine) (40). Furthermore, we saw no difference in the living number of bacterial cells between treatments and bacterial abundances were at very low numbers. Thus, it could be that the bacterial biomass was too low at the beginning of the experiment when prey was added. Supporting this idea, the open pitchers (positive control) showed higher hydrolytic enzyme activity later in the season, in addition to higher pitcher fluid pH and DNA concentration. These results align with what we expect in the natural environment where pitchers capture prey over time. To mimic the natural prey capture, a future experiment might introduce multiple nutrient pulses over time.

We saw differences in the pH of the pitcher fluid, driven by the ant treatment. Ants in the Formicidae family produce formic acid (the most basic carboxylic acid) as a poison but which also has significant bactericidal properties (41, 42). The presence of this acid in the ant treatment survived the autoclaving process (43) and likely contributed to the low pH in the pitcher fluid, although we do not see any effect on the number of living cells compared to the other treatments. In the United States, ants are the dominant prey in *S. purpurea* populations along the southeast range of the plant. The pH of the fluid could be an additional factor contributing to the selection and functioning of microbes assembled in pitchers. This is supported by our finding that pitchers from the ant treatment having consistently higher differential abundances of ASV 44 in the genus *Mucilaginibacter* (Fig. 5). Multiple species from this genus have been isolated from acidic *Sphagnum* peat bogs and are known to be acid-tolerant (44). Likewise, pitchers in the ant treatment had a high differential abundance of ASV 84 in the genus *Acidisoma*, which is often acidophilic (45, 46).

Finally, we can infer some measure of microbial function based on the diversity and magnitude of different carbon substrates used by the bacterial communities. Interestingly, despite the low pH of the ant treatment, we did not find those communities to be more efficient at metabolizing carboxylic acid substrates, over the four substrates that showed the largest differences between treatments, the fly treatment had increased utilization of three of them (compared with ant and/or beetle). The fly treatment showed the largest differences between the ant/beetle treatment for the amino acids—glycyl glutamic acid and threonine—possibly due to the fly treatment having the highest average percent protein of the three treatments (Fig. 1). The bacteria in the beetle and ant prey treatments showed increased metabolism of lactose; although the direct mechanisms for this are unclear, it could be that the increased metabolism of lactose is a result of microbes with unique machinery for hydrolyzing disaccharides into smaller monomers, like in lactose and other common carbohydrates. Microbial communities that assemble on certain types of substrates may exhibit resource specialization and nutritional preferences (47), although the observed patterns could arise from a variety of independent mechanisms including priority effects, microbial interactions, and spatial partitioning (48–50).

### Small changes in microbial community composition

Microbial communities within our three treatments were taxonomically diverse, spanning many Phyla, Families, and Genera. This pattern is consistent with the literature, which shows that functions related to decomposition, such as the expression of hydrolytic enzymes, can be expressed by a diverse range of bacteria and protozoa (51, 52). Interestingly, we found that the prey treatments with the highest ASV richness (ant and beetle) were not the ones that decomposed the fastest. This result contrasts with the well-known positive biodiversity-ecosystem functioning relationship (53, 54) and supports studies showing that diversity alone does not inform all aspects of community or ecosystem-level functioning (55–58). In fact, bacterial richness typically decreases during the decomposition of animal cadavers, likely due to the increase in taxa more specialized to the available substrates (59, 60). Supporting hypothesis 3, we observed differences in beta diversity between our treatments based on unweighted UniFrac, which takes into account the presence and absence of different taxa while accounting for phylogenetic distance (61), and thus gives more weight to rare taxa than a quantitative metric. This is consistent with empirical studies in other systems that find differences in beta diversity, sometimes driven by rare taxa (60) and often related to differences in decomposition, especially when microbial taxa differ in their functional characteristics (16).

Overall, there were many shared taxa between our treatments, likely due to the dispersal of microbes via rainwater, movement of protozoa, and through the air. Community composition across different insect prey treatments differed less than we had expected. Despite this, we did find some differentially abundant taxa among our three main treatments. As previously noted, some taxa that were relatively more abundant in pitchers from the ant treatment were from groups known to grow in more acidic conditions. Some taxa with higher differential abundances in pitchers with the fly treatment are from groups known to degrade insect material, such as ASV 40 from the genus *Chryseobacterium*, which contains species that degrade chitin and lipids (62, 63) and even have plant growth-promoting properties (64). Although we do not know the specific functional differences of the strains that make up key ASVs, our results support the idea that despite overall similarities in community composition, the presence of low-abundance, rare taxa has important functional implications for microbial communities (60, 65). Beyond treatment differences, we also see patterns in microbial community composition across time, likely driven by succession. The successional patterns in both composition and function can inform how microbial communities move through functional space over time (66).

### Conclusions

We investigated the relative importance of arthropod detrital type and quality on decomposition, microbial function, and microbial community composition. We find that detrital type and quality affect decomposition rates, largely determined by the proportion of exoskeleton, lipids, and protein in the prey biomass. Overall, arthropod detritus provides essential nutrients to microbial decomposer communities, with different prey influencing microbial function and the surrounding habitat in a variety of ways including hydrolytic enzyme activity and differences in fluid pH. This study broadens our understanding of how detrital inputs affect microbial communities and their nutrient cycling functions, supplementing other decomposition studies and providing a link between the plant and carrion decomposition literature.

## MATERIALS AND METHODS

### Sites and experimental design

This experiment was conducted at a high-altitude alpine bog in Switzerland (Les Mosses, LM, elevation 1,400 m) from June to August 2022. Insect prey came from three sources: they were either (i) caught locally at the LM site (2), collected at a low elevation site, Champ Buet, which is a restored aquatic wetland (fen) where pitcher plant populations are well-established (CB, elevation 600 m) in Switzerland, or (3) purchased as living insects online (ant, beetle, and fly, Table S1). Using a randomized block design, five plant blocks were selected at LM (Fig. 1) where each block consisted of many plants growing together. Each selected plant block was at least 1 m away from another block. Within each block, seven sterile, unopened leaves (pitchers) were selected and tagged with a zip-tie label. In total, thirty-five plants were selected and randomly assigned to one of seven groups (five plants in each group). Experimental groups included the addition of the following: autoclaved ants, autoclaved crickets, autoclaved flies, autoclaved mixed prey (LM local caught), and autoclaved mixed prey from low elevation sites (CB non-local caught). Justification for the decision for which insects to select (Diptera, Coleoptera, and Hymenoptera) was based on the topmost abundant insect prey type within Swiss populations of *S. purpurea* (33). Control groups included five plants with no prey addition (with prey exclusion bags) and five plants left unbagged with no prey addition (allowed to catch prey naturally). On day zero, unopened pitchers from each plant were manually opened (as aseptically as possible). Pitchers within each plant block were randomly assigned to treatment groups and were generally the same size and age. The pitcher was filled with 10–20 mL of sterile distilled water (depending on pitcher size) and one prey bag was added. The entire pitcher was covered with an autoclaved fine mesh bag (prey exclusion bag) and secured at the base with a zip tie. The prey exclusion bag allowed the dispersal of microbes and other small organisms like rotifers, oribatid mites, and protozoan but restricted the movement of insects into the pitcher.

Pitcher fluid samples were collected weekly for 7 weeks from June 2022 to August 2022. The prey exclusion bag was carefully removed from each plant; using gloved hands cleaned with 70% ethanol and a sterile pipette, the pitcher fluid was mixed 10 times, and a 2 mL aliquot was removed and stored in a sterile centrifuge tube. Then, the sample volume was replaced with sterile, nuclease-free water. In the field, 1.5 mL of pitcher fluid was centrifuged (Eppendorf, 5415C) at 5000 × *g* for 8 minutes, the supernatant was discarded, and 300 µL of Zymo DNA/RNA shield was added. The tubes were placed on ice during transport back to the lab and then stored at −20°C until DNA extraction and sequencing. The other 500 µL was transported back to the lab on ice and was processed the same day for microbial functional analysis, including quantification of chitinase and protease activity, Biolog EcoPlate carbon substrate use profiles, flow cytometry for bacterial cell count, protozoan community composition, pitcher fluid color, and pH. Pitcher fluid color was determined qualitatively, and pitcher fluid pH was measured using a 20-µL drop of well-mixed pitcher fluid on MQuant pH indicator strips (pH2.0–9.1, 1.09584.0001, Supelco, Bellefonte, PA, US) on days 7, 14, 21, 28, 35, 42, and 49. At the final sampling point (day 49), we collected each pitcher from the field by clipping it at the base and transporting it back to the lab on ice. Morphometrics were recorded for each pitcher including length, width, shoulder, aperture, and wet biomass. The pitchers were then dried at 75°C for 48 hours and re-weighed to record dry biomass (grams).

### Prey treatments

Insects were captured (on June 25^th^, 2022, at CB and June 26^th^, 2022, at LM) using a sweep net that had been sanitized with 70% ethanol. Bog sites were walked at a consistent pace while making brisk sweeps in a continuous figure-eight motion with the net. This process was continued for 10 minutes per site. The collected insects were deposited into a clean plastic bag and stored on ice for 3 hours until they could be stored at −20°C. Within 2 days, the insects were sorted by Order, counted, and weighed. Prey bags were constructed from nylon tea bags trimmed to 8 cm × 5 cm. Prey bags were made by weighing 0.053 +/− 0.001 g of dried prey into nylon tea bags. The mass of the empty bag and prey was recorded separately. Prey was kept frozen until added to the prey bag, heat sealed, and autoclaved at 121°C for 15 minutes. Bags that contained mixed prey got balanced numbers of insect Orders as representative of the captured prey in each community. A subset of each insect type was weighed and stored for future nutrient analysis. The prey bags were weighed at the beginning and end of the experiment to quantify the total insect biomass lost through decomposition (67). Nutrient composition of prey (total protein, lipids, and exoskeleton determination) was quantified according to Cuff et al. MEDI protocol (25). We quantified the gravimetric lipid content of each insect type using a 1:12 chloroform methanol extraction (25). Exoskeleton determination also followed Cuff et al. (25). Briefly, samples were dried at 65°C for 48 hours and weighed; insects were lightly cracked with a glass rod and soaked in 0.1M NaOH, after which they were rinsed with DI water, allowed to redry, and weighed. Protein content for insects was quantified using a Pierce Modified Lowry protein colorimetric assay (cat #23240, Thermo Fisher Scientific), which was found to be a close proxy for amino acid analysis (26). The three main types of prey (ant, beetle, and fly) had different percentages of exoskeleton, lipids, and proteins (Fig. 1; Fig. S1). Beetles and ants had the highest percentage of exoskeleton compared with flies, whereas flies had the highest percent lipids (Fig. 1; Fig. S1).

### Quantifying microbial function

Chitinase and protease activity were quantified each week for each sample of pitcher fluid using fluorometric assays developed for black 96-well microplates (68). Changes in fluorescence emission were measured every 5 minutes for the duration of a 60-minute kinetic run using a microplate reader (Hidex Sense, FIN-20520 Turku, Finland). Chitinase activity was measured using 200 µL of sample (unfiltered pitcher fluid) and 50 µL of substrate (0.86 mM 4-Methylumbelliferyl N,N′-diacetyl-β-D-chitobioside in 50 mM Tris-HCl pH 8.0 with 0.1% bovine serum albumin) as described in (40). Standards of 4-methylumbelliferone were made from 0 to 1 µM concentrations. Protease activity was measured using 50 µL of sample added to 25 µL of 50 mM Tris-HCl pH 7.5 and 75 µL of substrate (2 mM L-leucine-7-amido-4-methylcoumarin hydrochloride in nanopure water) (40). Standards of 7-amino-4-methylcoumarin were made from 0 to 1,000 nM concentrations. In both cases, plates were read at 355 nm excitation and 460 nm emission. Using the standard curves for each assay, fluorometric readings were converted to product concentrations and plotted along time to get the linear enzymatic rate for each sample.

The community-level carbon substrate use was measured for each sample every other week (days 7, 21, 35, and 49) of the experiment using Biolog EcoPlates (69). EcoPlates are carbon substrate-embedded 96-well plates that show differential absorbance based on microbial consumption of the substrates. These 31 substrates can be classified into six broad categories: amino acids, amines, carbohydrates, carboxylic acids, polymers, and phenolic compounds (70). EcoPlates were allowed to incubate in the dark at room temperature, absorbance readings were collected every 24 hours until a maximum color change had been observed (72 hours). The changes in color were quantified by reading the absorbance using a plate reader (previously mentioned) at a wavelength of 590 nm. Water was used as a control blank and subtracted from each absorbance reading prior to analysis.

Bacterial density for each pitcher sample was quantified using flow cytometry (BD Accuri C6, BD Biosciences, San Jose, CA). Living and dead bacteria were differentially stained using SYBR Green I (Thermo-Fisher Scientific Inc., Waltham, MA) and Propidium Iodide, run limits set to 5 µL, flow rate set to 35 µL/min, and FL1-H threshold set at 1,000 per sample and analyzed using CFlow Software. Samples were gated based on standard gates used for differential staining (71). Proportions of living and dead bacterial cells were calculated for each sample.

### Microbial community composition

Because of limited resources, we chose to only explore microbial community composition at three time points, we chose day 7 because this was our first sampling day, we chose day 14 because we expected most of our microbial functional dynamics to occur early in successional time based on other research (72, 73), and we chose day 35 as our final time because we expected community composition to have stabilized by this point. DNA was extracted and amplified for 16S rRNA amplicon sequencing for all samples from day 7, day 14, and day 35 to identify microbial community composition using the DNAdvance Genomic DNA Isolation Kit (Beckman Coulter A48705) using 750 µL of sample. Samples were bead beaten in lysis buffer at 2,400 rotations per minute (RPM) for 10 minutes and then incubated at 55°C shaking (150 RPM) overnight before proceeding with the extraction (halving all the reagents but following the protocol per the manufacturer’s directions), each 96-well plate included one negative control. DNA was quantified using AccuClear Ultra HS dsDNA kit (Biotium #31028) and fluorometer. Library preparation and sequencing was conducted by the Environmental Sample Preparation and Sequencing Facility at Argonne National Laboratory. Sequencing for metabarcoding samples was performed on a 151 bp × 12 bp × 151 bp MiSeq run targeting the V4 region of 16S rRNA using the 515F and 806R primers.

The 16S rRNA gene sequences were processed in QIIME2 (2023.5), demultiplexed using no-golay error correction and quality filtered to remove reads with a mean score less than 20, and trimmed to the sequence length of 150 base pairs. The DADA2 (74) module was used to denoise sequences and generate amplicon sequence variants (ASVs) with a median length of 253 base pairs. Taxonomy was assigned using the classify-sklearn method, which is a Native Bayes classifier, and a pre-trained classifier made with SILVA v. 138 database containing 99% ASVs from 515F/806R region (75). The phylogenetic tree was built using multiple-sequence alignment via multiple sequence alignment program (MAFFT) and phylogenetic reconstruction via FastTree, both via QIIME2 plugins. Across 102 samples (98 pitcher fluid samples and four negative controls), DADA2 generated 1321 ASVs. Using the *decontam* R package (76), 37 contaminant ASVs were identified and discarded. The *decontam* method “prevalence” was used, which identified ASVs based on their presence and abundance in our negative controls. Data were quality filtered to remove non-prokaryotic ASVs (including removing taxa classified as mitochondria or chloroplasts), negative controls, and only include observations with at least 10 sequences and samples with at least 1,000 sequences, resulting in 98 pitcher fluid samples (day 7, day 14, and day 35) with a cumulative 456 distinct ASVs, with 26,393 mean reads per sample (min reads/sample = 4,431; max reads/sample = 48,534).

To measure protozoan community composition, an aliquot of 50 µL pitcher fluid was used to determine the presence or absence of and (when possible) to identify the protozoan species with a compound microscope (Magnification = 200X) (77). We recorded the presence or absence by doing 7–10 passes per coverslip covering the entire area (24 × 24 mm). The mesh bags covering the plants had spaces generally large enough for protozoa and rotifers to fit through, but there may be some limit to their dispersal not accounted for.

### Statistical analysis

All statistical analyses were conducted in R version 4.3.2 (78). To make inferences about the effects of prey type on microbial community function and composition, we measured many microbial community traits (hydrolytic enzyme activity, carbon substrate use, bacterial density, protozoan community composition, and bacterial community composition). We developed Bayesian GLMMs to estimate the effect of different prey types on decomposition and microbial function. When the distribution of the data was positive and continuous, we used a gamma distribution with a log-link; when the response was proportions bounded between zero and one, we used a beta distribution; finally, for discrete count data, we used a negative binomial distribution in our models. The models were fitted using the R package *brms* (79), which uses the Hamiltonian Monte Carlo (HMC) Markov chain Monte Carlo (MCMC) algorithm implemented in Stan to estimate the parameter coefficients. The categorical variables were re-leveled; hence, the “fly” or “local” prey treatment was the baseline. Default uninformative priors were used, convergence and mixing of chains and unimodality in posterior predictions were visually assessed, and all R-hat values were equal to 1.0 (80). To account for repeated measures, time was included as a fixed effect when sampling had occurred in pitchers over time, and plant block was included as a random intercept. In the cases when the model would not converge, the random effect was removed. The model fit was evaluated using the posterior predictive check function in the *brms* package (79).

We compared EcoPlate carbon substrate use between treatment groups and through time using permutational multivariable analysis of variance (PERMANOVA) (37), and the *adonis2* function in vegan based on Bray-Curtis dissimilarities [adonis2(y ~ treatment + time, by =”margin)]. Additionally, we did a pairwise adonis using vegan to examine which treatments were different from each other [pairwise.adonis(y ~ treatment)]. For microbial community analysis, samples were rarefied to minimum sample depth (4,431 reads, no loss of samples) using the *rrarify* function in vegan (81). Dispersion was calculated using the *betadisper* function in *vegan*. We compared bacterial communities between treatment groups and through time using a PERMANOVA based on unweighted UniFrac dissimilarities (61). Additionally, we did a pairwise adonis using vegan to examine which treatments were different from each other. We examined which ASVs were differentially abundant across treatment groups (formula = ancombc2(data = tse, assay_name = "counts," tax_level = NULL, fix_formula = "treatment + day," p_adj_method = "fdr," pseudo_sens = TRUE, prv_cut = 0.07, group = "treatment," alpha = 0.05, n_cl = 3, verbose = TRUE, global = TRUE, pairwise = TRUE) using ANCOMBC2 with the fly treatment set as the baseline (82). We compared protozoan community composition between treatment groups using a PERMANOVA based on Jaccard dissimilarities.

## Data Availability

Scripts and data associated with this manuscript are available at: https://github.com/jessibernardin/prey_type_microbial_function. The raw reads for 16S rRNA amplicon sequencing have been deposited in the National Center for Biotechnology Information (NCBI) under the project number PRJNA1071685.

## SUPPLEMENTAL MATERIAL

Supplemental material aem.00394-24-s0001.docx Figures S1 to S7; Tables S1 to S3.

ASM does not own the copyrights to Supplemental Material that may be linked to, or accessed through, an article. The authors have granted ASM a non-exclusive, world-wide license to publish the Supplemental Material files. Please contact the corresponding author directly for reuse.
