# Supplementary material for: Arthropod prey type drives decomposition rates and microbial community processes
Source: Appl Environ Microbiol. 2024 Jun 25;90(7):e00394-24. doi: 10.1128/aem.00394-24 (PMC11267907; doi:10.1128/aem.00394-24)
Supplement: Supplemental material — Figures S1 to S7; Tables S1 to S3. [file aem.00394-24-s0001.docx]

Supplementary Results for:

Arthropod prey type drives decomposition rates and microbial community processes

Jessica R. Bernardin^1^, Sarah M. Gray^2^, Leonora S. Bittleston^1^

Corresponding author: Jessica R. Bernardin: jessicabernardin@boisestate.edu

This PDF file includes Supplementary Results:

Figures S1 to S7

Tables S1 to S3


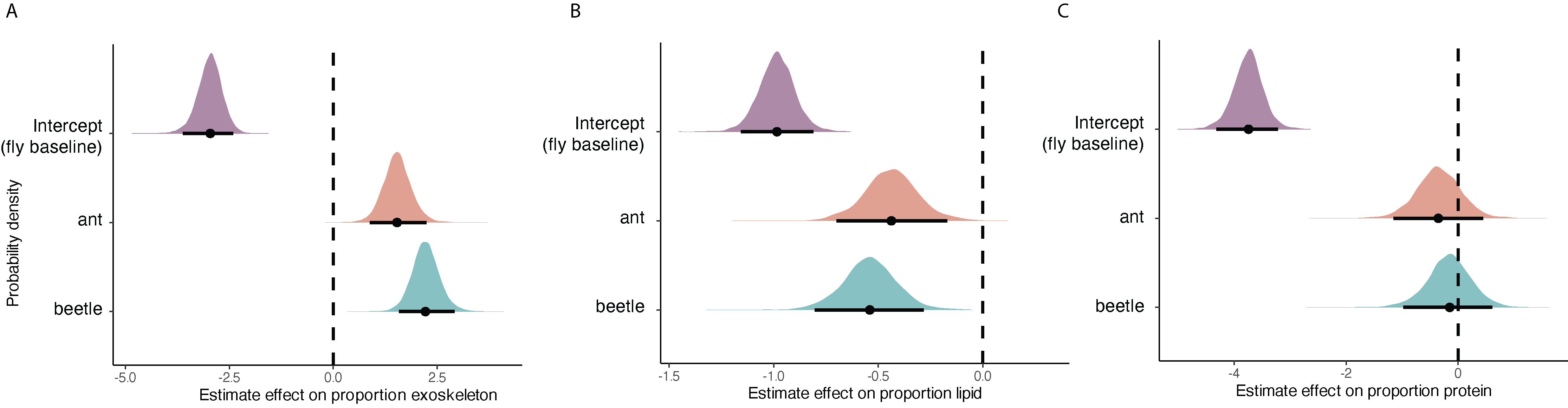


**Figure S1.** The posterior estimate of the proportion of nutrients for ant, beetle and fly prey. **A)** The posterior estimates of the effect of prey type on the proportion of dry biomass that is exoskeleton compared to fly (baseline, 0). **B)** The posterior estimates of the effect of prey type on the proportion of dry biomass that is lipids compared to fly (baseline, 0). **C)** The posterior estimates of the effect of prey type on the proportion of dry biomass that is protein compared to fly (baseline, 0). The points represent the median estimate, and the black bars represent the 95% credibility intervals around those estimates.


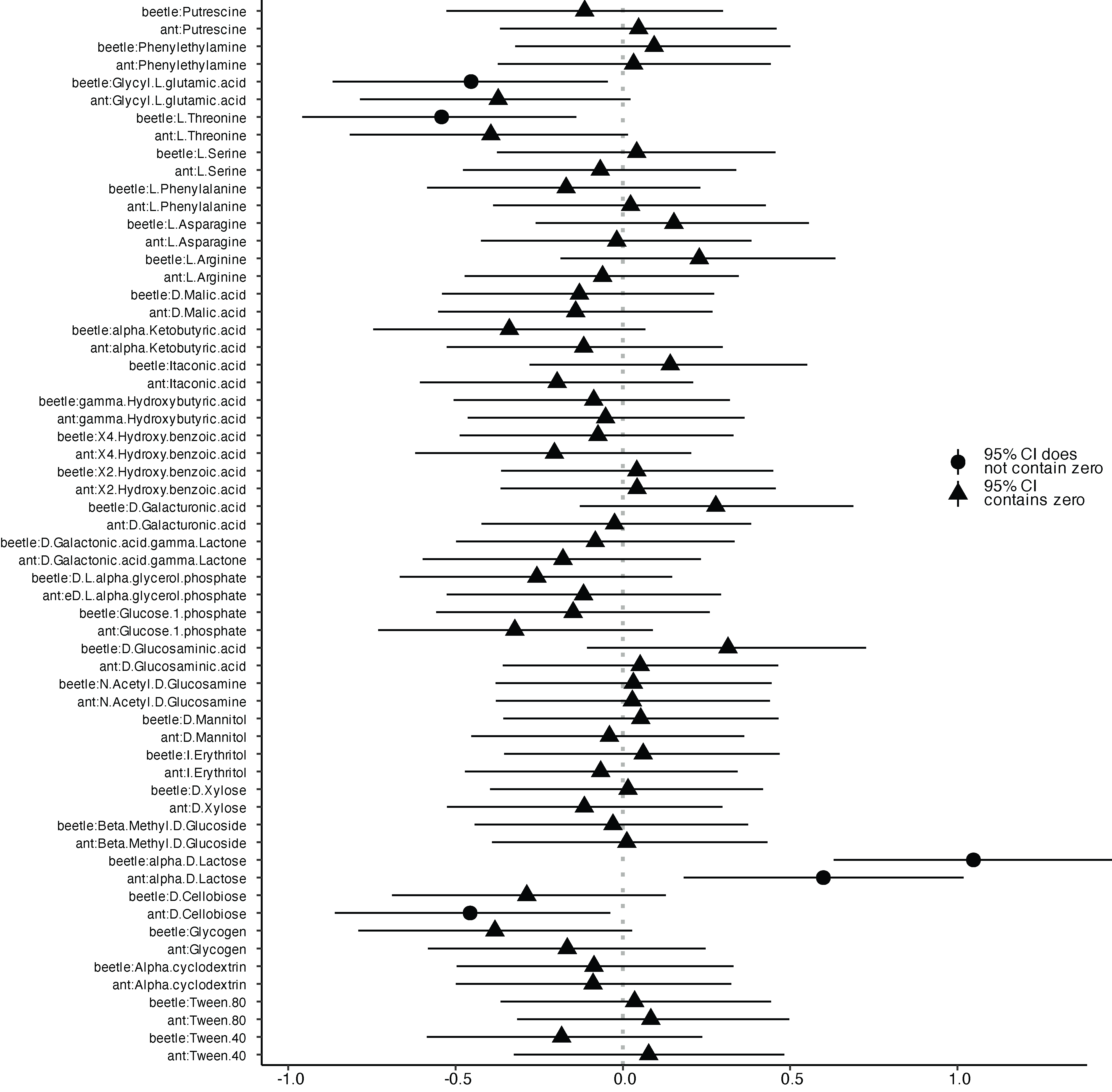


**Figure S2.** The posterior estimate of the effect of prey and EcoPlate carbon substrate type on absorbance (metabolism of that particular substrate based on color change of tetrazolium dye). The posterior estimates based on a normal distribution, circle estimates represent treatment effects that are different from fly (95CIs don’t cross zero), triangles represent estimates and credibility intervals that do cross zero. The points and triangles represent the median estimate, and the black bars represent the 95% credibility intervals around those estimates.


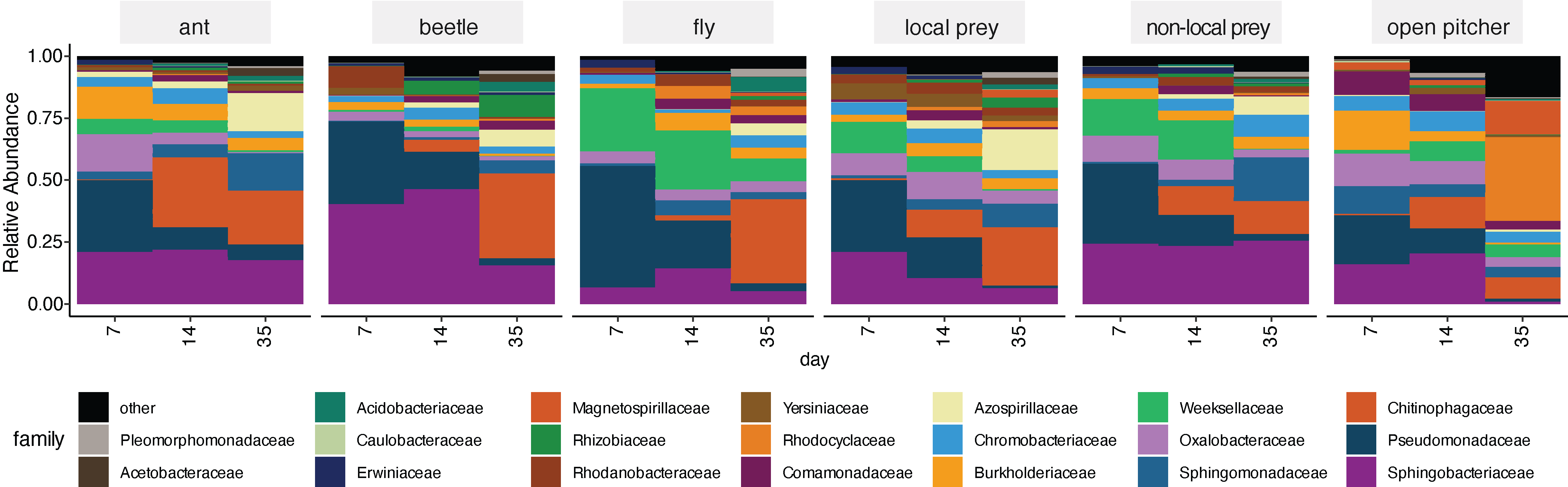


**Figure S3.** Bacterial relative abundance showing **t**axonomic compositional differences between the arthropod treatments identified from 16S rRNA analysis of pitcher fluid samples. Relative abundance to top 20 most abundant families at day 1, 14, and 35 for the five prey treatments and the positive control (open pitcher).


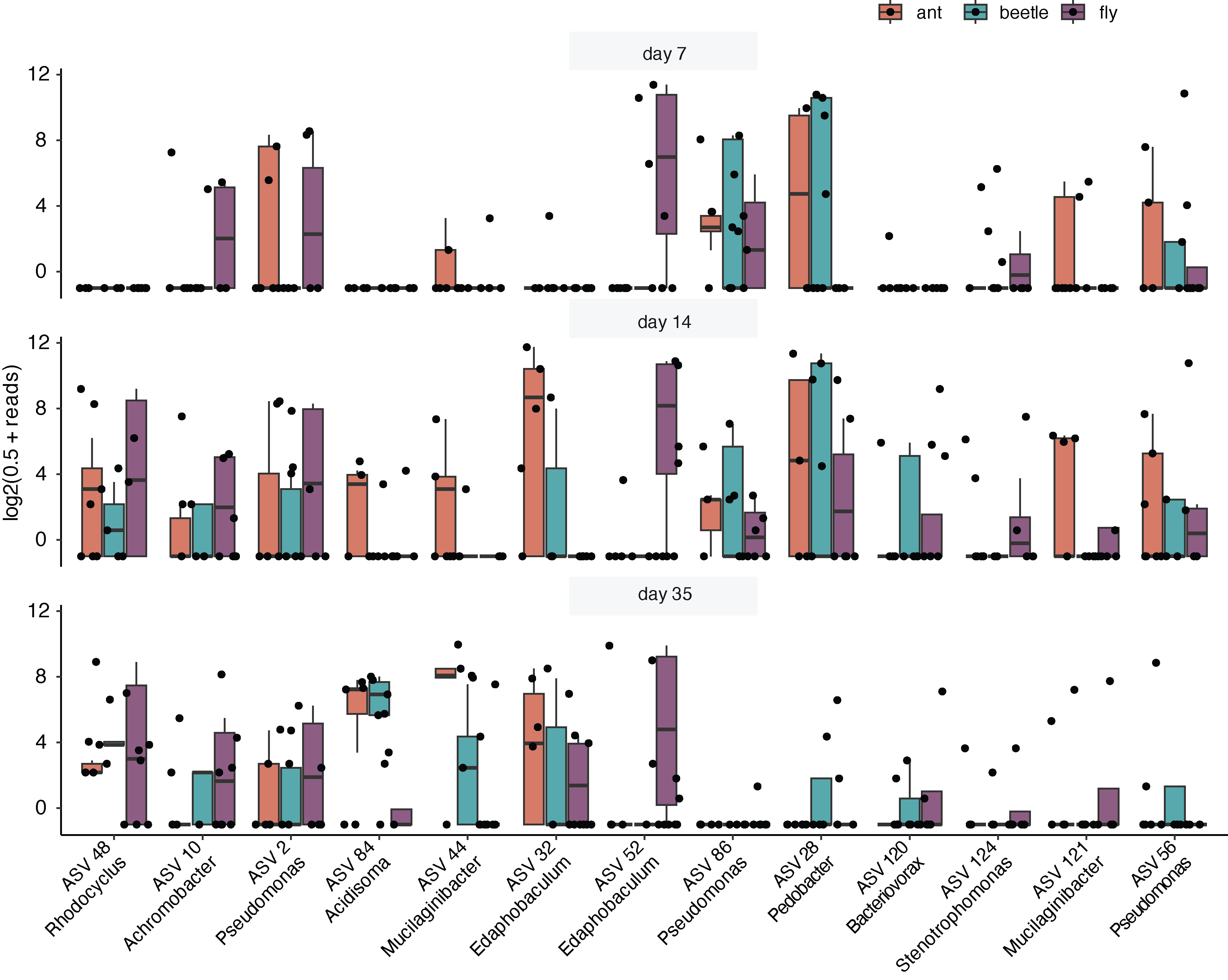


**Figure S4.** Thirteen differentially abundant ASVs at day 7, 14, and 35. Reads were transformed by log2(0.5 + reads). In all cases, boxplots represent the interquartile range (IQR) of the counts for each sample in each treatment, the whiskers extend 1.5 times the IQR, with the horizontal bars representing the medians. ASV number and Genera represented on the x-axis.


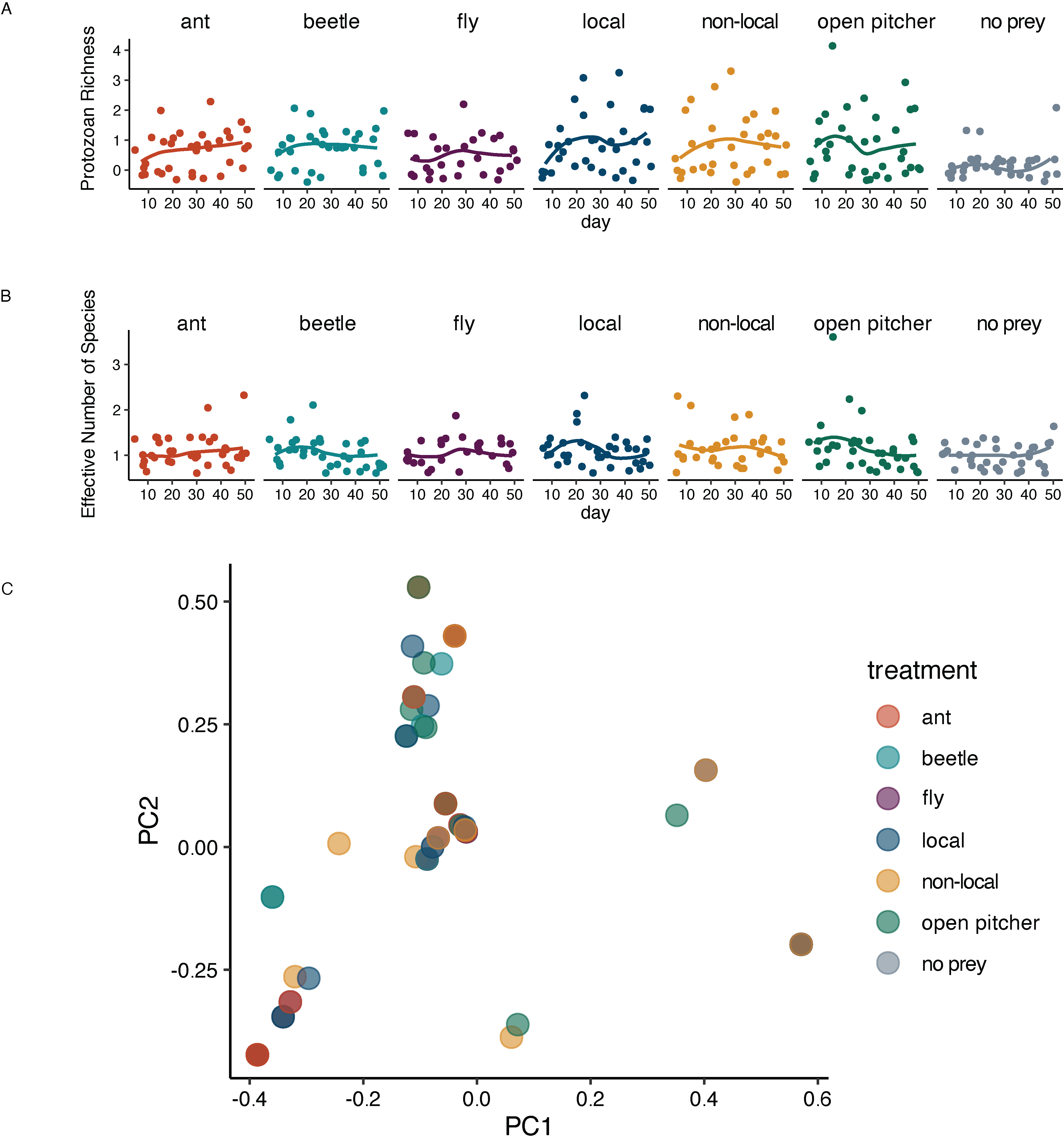


**Figure S5.** Protozoa community composition weekly measurements for seven weeks. **A)** Protozoan richness (Hill number 0) for all the prey treatments and controls. **B)** Protozoan effective number of species (Hill number 1) for all the prey treatments and controls. Each colored point represents the raw data, and polynomial lines of best fit are presented. **C)** Principal coordinates analysis (PCoA) of protozoa community composition in the pitcher fluid samples over seven weeks, k=2, distances based on Jaccard dissimilarities (PERMANOVA; R^2^= 0.05231, F_6,103_=1.0309, p=0.074). The individual samples are color coordinated based on prey type or control.


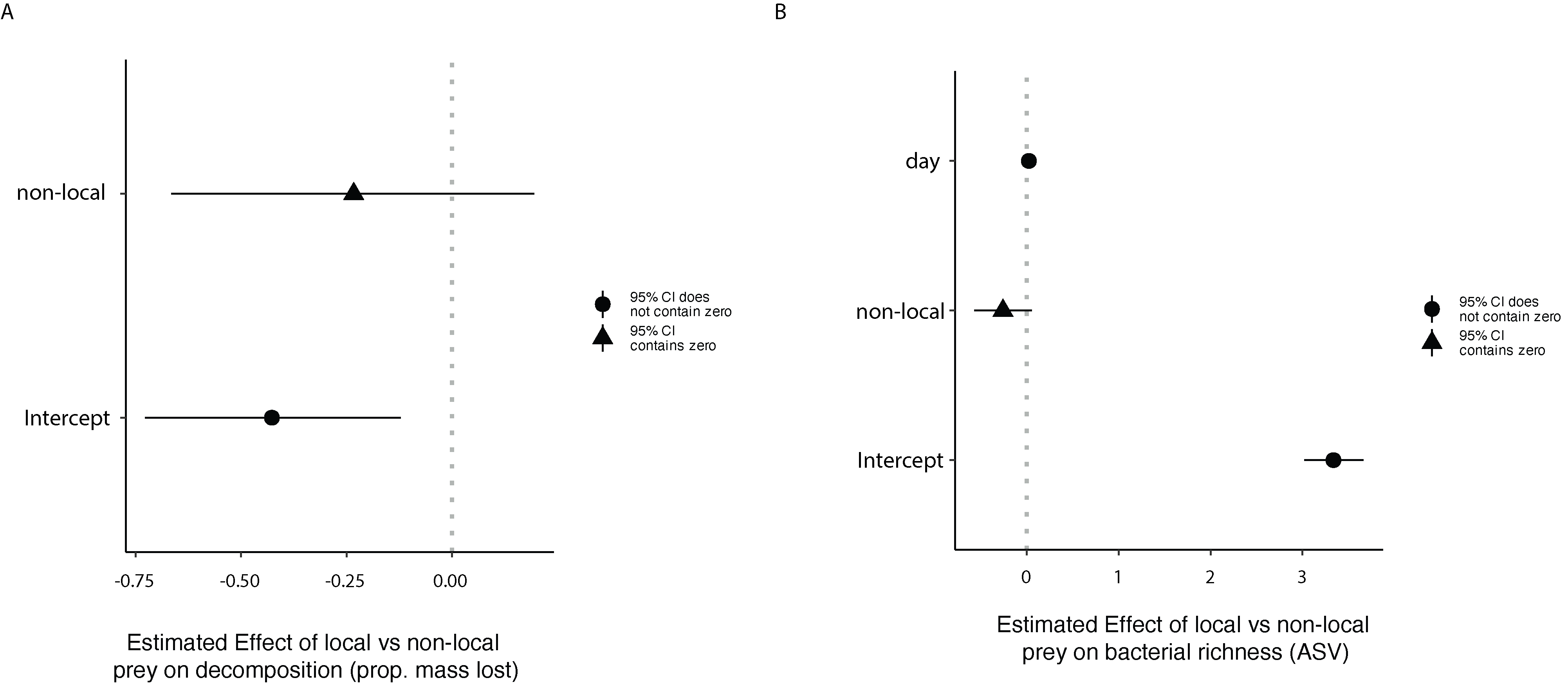


**Figure S6. A)** Posterior estimates for the effect of local vs non-local prey on decomposition. **B)** Posterior estimates of the effect of local vs non-local prey and time on bacterial richness (ASV). The posterior estimates based on a negative binomial distribution, circle estimates represent treatment effects that are different from local prey (95CIs don’t cross zero), triangles represent estimates and credibility intervals that cross zero. The points and triangles represent the median estimate, and the black bars represent the 95% credibility intervals around those estimates. There is no effect of prey collection site (non-local prey) on decomposition or bacterial richness compared to local prey.


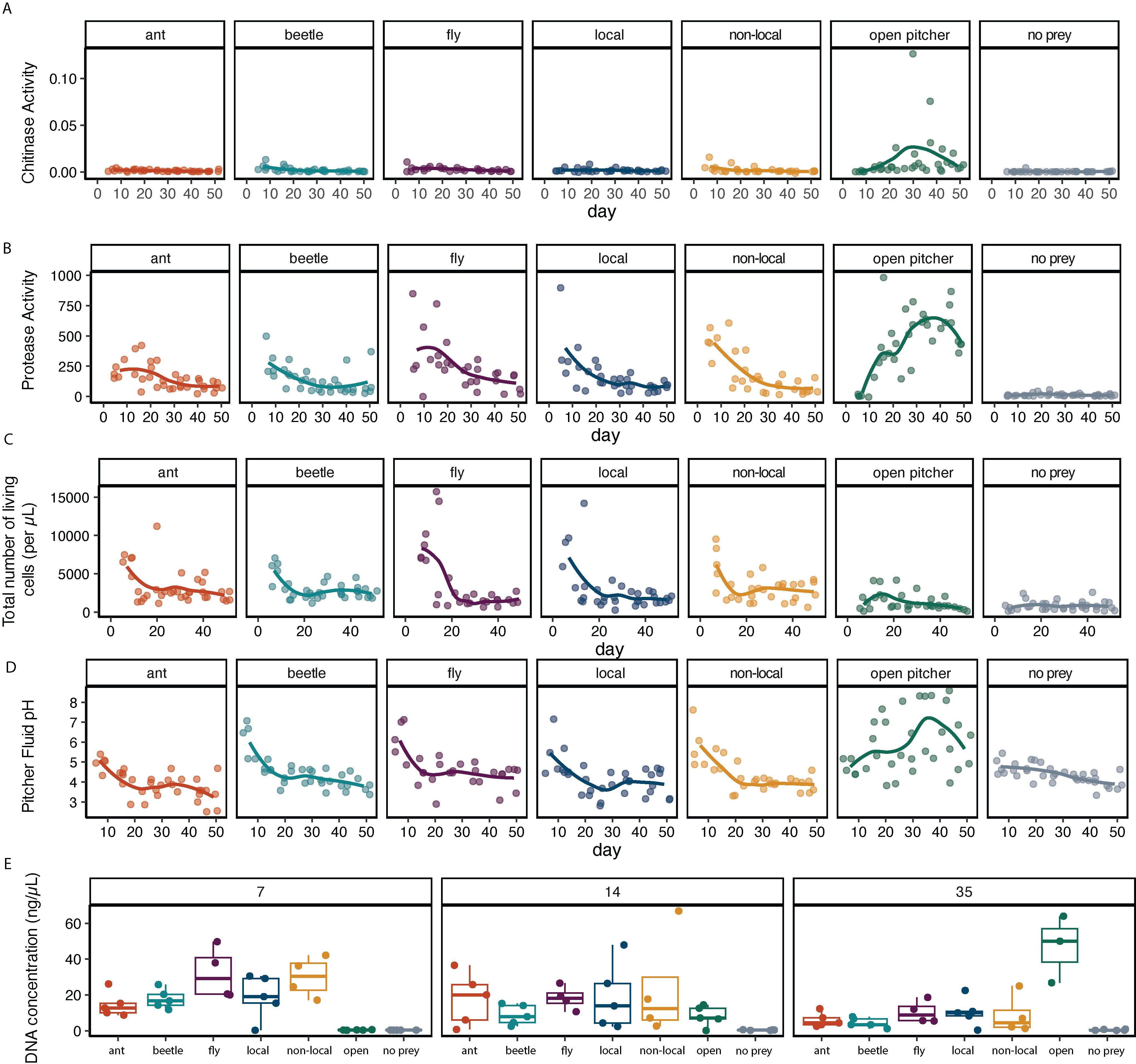


**Figure S7.** Microbial functions across all treatments and controls. **A**) Chitinase activity (µg chitin substrate/min) for each sample across seven weeks. **B**) Protease activity (ng leucine substrate/min) for each sample across seven weeks. **C**) Number of living bacterial cells in one microliter of pitcher fluid for each sample across seven weeks. **D**) Pitcher fluid pH for each sample across seven weeks. **E**) Concentration of genomic DNA (ng/µL) for each sample at day 7, 14, and 35. For **A-E**, each colored point represents the raw data, in **A-D** polynomial lines of best fit are presented, in **E** boxplots represent the interquartile range (IQR) of the concentration of DNA in each treatment, the whiskers extend 1.5 times the IQR, with the horizontal bars representing the medians.

**Table S1.** List of the three main types of arthropod prey, scientific name, common name, and purchase information.

| Insect Scientific Name | Common Name | Company | URL |
| --- | --- | --- | --- |
| *Pogonomyrmex barbatus* | Red Harvester Ant | Insect Lore | https://www.amazon.com/dp/B08WPNDQVW/ref=pe_386300_440135490_TE_item |
| *Alphitobius diaperinus* | Black Cleaner Beetles | Amazon | https://www.amazon.com/dp/B071GJQWP2/ref=pe_386300_440135490_TE_item |
| *Hermetia illucens* | Black Soldier Fly | Symton | https://symtonbsf.com/products/black-soldier-fly-pupae?_pos=1&_sid=31adcc186&_ss=r |

**Table S2.** EcoPlate carbon substrate profiles pairwise PERMANOVA results between the ant, beetle, and fly prey treatments, pairwise.adonis2(com ~ treatment, data = com_meta, strata = 'week').

|  |  | degrees of freedom | sum of squares | R^2^ | F | p value |
| --- | --- | --- | --- | --- | --- | --- |
| ant vs beetle | treatment | 1 | 0.083 | 0.06312 | 2.5601 | 0.003 |
|  | Residual | 38 | 1.232 | 0.93688 | NA | NA |
|  | Total | 39 | 1.315 | 1 | NA | NA |
| ant vs fly | treatment | 1 | 0.06998 | 0.04815 | 1.7705 | 0.043 |
|  | Residual | 35 | 1.38342 | 0.95185 | NA | NA |
|  | Total | 36 | 1.45340 | 1 | NA | NA |
| beetle vs fly | treatment | 1 | 0.11077 | 0.09466 | 3.6597 | 0.002 |
|  | Residual | 35 | 1.05932 | 0.90534 | NA | NA |
|  | Total | 36 | 1.17009 | 1 | NA | NA |

**Table S3.** Beta diversity based on unweighted UniFrac distances. Pairwise PERMANOVA results between ant, beetle, and fly prey treatments, pairwise.adonis2(wu.dist.16s ~ treatment, data = data_merge2, strata = 'day').

|  |  | degrees of freedom | sum of squares | R^2^ | F | p value |
| --- | --- | --- | --- | --- | --- | --- |
| ant vs beetle | treatment | 1 | 0.1545 | 0.03642 | 1.0582 | 0.156 |
|  | Residual | 28 | 4.0880 | 0.96358 | NA | NA |
|  | Total | 29 | 4.2425 | 1 | NA | NA |
| ant vs fly | treatment | 1 | 0.2260 | 0.05416 | 1.4316 | 0.043 |
|  | Residual | 25 | 3.9457 | 0.94584 | NA | NA |
|  | Total | 26 | 4.1717 | 1 | NA | NA |
| beetle vs fly | treatment | 1 | 0.1809 | 0.04082 | 1.064 | 0.158 |
|  | Residual | 25 | 4.2508 | 0.95918 | NA | NA |
|  | Total | 26 | 4.4317 | 1 | NA | NA |
